# Mechanisms for an effect of acetylcysteine on renal function after exposure to radio-graphic contrast material: study protocol

**DOI:** 10.1186/1472-6904-12-3

**Published:** 2012-02-03

**Authors:** Euan A Sandilands, Sharon Cameron, Frances Paterson, Sam Donaldson, Lesley Briody, Jane Crowe, Julie Donnelly, Adrian Thompson, Neil R Johnston, Ivor Mackenzie, Neal Uren, Jane Goddard, David J Webb, Ian L Megson, Nicholas Bateman, Michael Eddleston

**Affiliations:** 1 National Poisons Information Service (Edinburgh), Royal Infirmary of Edinburgh, Edinburgh, UK; 2 Clinical Pharmacology Unit, University/BHF Centre for Cardiovascular Science, University of Edinburgh, The Queen's Medical Research Institute, Edinburgh, UK; 3 Wellcome Trust Clinical Research Facility, Royal Infirmary of Edinburgh, Edinburgh, UK; 4 Free Radical Research Facility, University of the Highlands & Islands, Inverness, UK; 5 Department of Cardiology, Royal Infirmary of Edinburgh, Edinburgh, UK; 6 Department of Renal Medicine, Royal Infirmary of Edinburgh, Edinburgh, UK

**Keywords:** Contrast-induced nephropathy, acetylcysteine, prevention, kidney, contrast media

## Abstract

**Background:**

Contrast-induced nephropathy is a common complication of contrast administration in patients with chronic kidney disease and diabetes. Its pathophysiology is not well understood; similarly the role of intravenous or oral acetylcysteine is unclear. Randomized controlled trials to date have been conducted without detailed knowledge of the effect of acetylcysteine on renal function. We are conducting a detailed mechanistic study of acetylcysteine on normal and impaired kidneys, both with and without contrast. This information would guide the choice of dose, route, and appropriate outcome measure for future clinical trials in patients with chronic kidney disease.

**Methods/Design:**

We designed a 4-part study. We have set up randomised controlled cross-over studies to assess the effect of intravenous (50 mg/kg/hr for 2 hrs before contrast exposure, then 20 mg/kg/hr for 5 hrs) or oral acetylcysteine (1200 mg twice daily for 2 days, starting the day before contrast exposure) on renal function in normal and diseased kidneys, and normal kidneys exposed to contrast. We have also set up a parallel-group randomized controlled trial to assess the effect of intravenous or oral acetylcysteine on patients with chronic kidney disease stage III undergoing elective coronary angiography. The primary outcome is change in renal blood flow; secondary outcomes include change in glomerular filtration rate, tubular function, urinary proteins, and oxidative balance.

**Discussion:**

Contrast-induced nephropathy represents a significant source of hospital morbidity and mortality. Over the last ten years, acetylcysteine has been administered prior to contrast to reduce the risk of contrast-induced nephropathy. Randomized controlled trials, however, have not reliably demonstrated renoprotection; a recent large randomized controlled trial assessing a dose of oral acetylcysteine selected without mechanistic insight did not reduce the incidence of contrast-induced nephropathy. Our study should reveal the mechanism of effect of acetylcysteine on renal function and identify an appropriate route for future dose response studies and in time randomized controlled trials.

**Trial registration:**

Clinical Trials.gov: NCT00558142; EudraCT: 2006-003509-18.

## Background

Radiographic contrast material has been used for over 70 years to enhance medical imaging in diagnostic and interventional procedures. While considered generally safe in healthy patients [1,2], it can lead to renal impairment particularly in the presence of co-morbidity [3]. Contrast-induced nephropathy (CIN) is typically defined as an increase in serum creatinine of 25% over baseline, or an absolute increase of 0.5 mg/dL (44.2 μmol/l), within 48 hours of contrast administration [1]. Although usually reversible, renal replacement therapy may be required in a small number of patients.

Contrast-induced nephropathy occurs in less than 2% of patients with normal renal function, but in up to 50% of those with pre-existing renal impairment and diabetes [1]. Other risk factors include congestive cardiac failure, age over 70 years, dehydration, and concurrent administration of nephrotoxic drugs [4,5]. With increasing utilisation of contrast during investigations such as coronary angiography, particularly in an ageing population with multiple co-morbidities, CIN has become a significant source of hospital morbidity and mortality [1,2,6]. Not only has it been reported as the third most common cause of in-hospital acute renal failure [7], patients with chronic kidney disease (CKD) who develop CIN following PCI demonstrate a 3-fold increase in hospital mortality compared to those without CIN (14.9% vs 4.9% in one study) [8].

### Mechanism

Injury to the renal medulla appears to be the primary problem in CIN, although the precise mechanisms involved are not well understood. Current hypotheses include disturbances in renal haemodynamics, an osmotic effect, and a direct toxic effect of contrast media on tubular epithelial cells [1,9,10]. The last of these may be a result of toxic free radical release occurring after contrast administration. Whether these mechanisms act separately or together to cause renal insufficiency is not clear. Administration of contrast leads to a biphasic haemodynamic change in the kidney, with an initial transient increase followed by a prolonged decrease in renal blood flow (RBF) [9]. Current prevention strategies therefore aim at maintaining urine flow and reducing oxidative stress [11]. This may be achieved through the use of intravenous fluids, a low dose of iso-osmolar contrast, withholding nephrotoxic drugs, and perhaps acetylcysteine via its vasodilatory and antioxidative properties [4].

Despite a paucity of randomised controlled trial (RCT) data, intravenous hydration pre- and post-contrast is widely accepted as beneficial in counteracting the effects of contrast through augmenting RBF and glomerular filtration rate (GFR) [4,6]. Hydration regimens are generally based on administration of ~1 mL/kg per hour, commencing 6-12 hours pre-contrast and continuing for 12 hours post-contrast [6,12,13]. While sodium chloride is most commonly recommended, sodium bicarbonate might further reduce the risk of CIN [14]. Such regimens, however, are not practicable in the out-patient setting.

Contrast agents are classified according to osmolality. This is important as agents with greater osmolality are more nephrotoxic [4]. First generation hyperosmolar (1500 - 1800 mOsm/kg) agents have gradually been replaced by low-osmolar (600 - 850 mOsm/kg), and subsequently, iso-osmolar (280 mOsm/kg) agents, both of which cause less CIN than first generation agents [4]. Any additional benefit offered by iso-osmolar over low-osmolar agents, however, is less clear [15-18]. In an RCT of 129 patients, Aspelin *et al. *[19] reported a significantly reduced incidence of CIN associated with the use of the iso-osmolar agent iodixanol compared to the low-osmolar agent iohexol [19]. However, this study did not control for the volume of contrast administered, an independent predictor for the development of CIN [20,21]. A more recent RCT reported no significant difference in the incidence of nephrotoxicity associated with iso- and low-osmolar agents [22].

### The role of acetylcysteine

Acetylcysteine is commonly given before contrast media in patients with renal impairment in an effort to minimise the risk of CIN. Use of this agent is attractive due to its wide availability, ease of administration, and low cost. However, despite extensive research, any benefit offered by acetylcysteine remains unclear. In fact, a CIN Consensus Working Panel reported in 2006 that "no adjunctive medical or mechanical treatment has been proved to be efficacious in reducing the risk of CIN", and specifically that "N-acetylcysteine is not consistently effective in reducing the risk for CIN" [11].

A systematic review called for a large multi-centre RCT to be conducted to address this research question [23]. However, RCTs to date have been conducted without detailed knowledge of the effect of acetylcysteine on renal function that would have guided the choice of outcome measure or regimen. A variety of doses (from around 7 to 200 mg/kg total doses) and routes of administration have also been used [23]. Therefore, there is first a need for a detailed mechanistic study of acetylcysteine use in patients with chronic kidney disease receiving contrast medium [24]. Such a study should inform any decisions regarding the most appropriate dose and route of administration of acetylcysteine.

Acetylcysteine possesses both vasodilatory [25] and antioxidative [4] properties and may be renoprotective via these mechanisms. Previous studies have assessed changes in serum creatinine following acetylcysteine administration. Serum creatinine, however, is not only an insensitive marker of altered renal function [26], but acetylcysteine itself may cause a reduction in serum creatinine independent of GFR [27]. Furthermore, if acetylcysteine offers renoprotection via vasodilatation, serum creatinine would not be the most appropriate marker. Finally, as highlighted in previous studies that have used a variety of doses (from around 7 to 200 mg/kg total doses) and routes of administration, the optimum dose and route of administration is not yet known [23].

We hypothesized that acetylcysteine may exert a renoprotective effect in CIN by a mechanism involving renal vasodilatation and/or its potential role as an antioxidant. To investigate this we took a structured 4-part approach to the question, using randomized controlled crossover studies to assess the effect of acetylcysteine on renal function in both normal and diseased kidneys, and the effect of contrast on normal kidneys, with and without acetylcysteine treatment. We also designed a parallel-group randomized controlled trial of patients with CKD stage III undergoing elective coronary angiography with and without acetylcysteine treatment. We believe this mechanistic study will enable us to control variables and interpret any role acetylcysteine plays in preventing CIN in CKD patients.

## Methods/Design

### Study design

**Figure 1:**
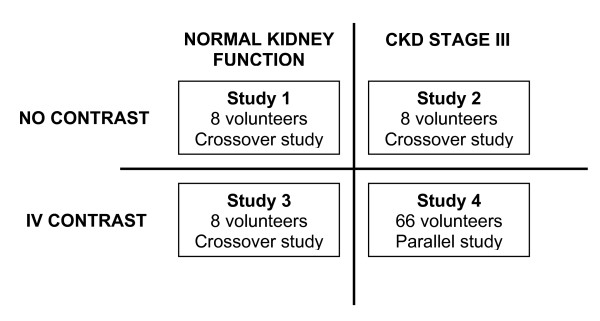
**4 way study design**.

The study is being performed simultaneously in four groups of participants (Figure 1). The local research ethics committee approved the protocol and written informed consent is attained from each participant before entering the study. Studies 1-3 are randomised, placebo-controlled, three-way, crossover human volunteer studies of eight participants. All studies are performed at the Wellcome Trust Clinical Research Facility, Royal Infirmary of Edinburgh.

Study 1 investigates healthy volunteers receiving oral acetylcysteine, intravenous (IV) acetylcysteine, or placebo; study 2 patients with CKD stage III receiving oral acetylcysteine, IV acetylcysteine, or placebo; and study 3 healthy volunteers receiving IV contrast plus either oral acetylcysteine, IV acetylcysteine, or placebo. Participants are randomised to receive all three treatments on three different occasions.

Study 4 is a randomised, placebo-controlled, three-way parallel group study in patients undergoing elective coronary angiography with sufficient studies being performed to obtain 66 completed studies. Participants in this study are randomised to one arm only, receiving oral acetylcysteine, IV acetylcysteine, or placebo.

Participants are followed up at 24 hrs and 72 hrs after acetylcysteine administration.

We are studying healthy volunteers in order to identify the effects of acetylcysteine and contrast on healthy kidneys. This should provide us with data to help interpret the studies in CKD patients. In addition, while healthy patients rarely get CIN after contrast administration, the currently used marker of CIN (raised serum creatinine) is a crude measure of renal dysfunction since GFR will only fall after RBF has been substantially reduced for a long period of time. It is likely that small sub-clinical changes will occur in healthy patients that will help illustrate how contrast and acetylcysteine affect the kidney [28]. Any changes in renal function that occur in the volunteers are expected to be transient.

### Subjects

All participants are non-smoking, male volunteers aged over 45 years with a body mass index (BMI) of 22-40 kg/m^2^. Exclusion criteria for studies 1 and 3 include clinically significant co-morbidity (heart failure, hypertension, diabetes mellitus, coagulopathy); thyroid disease, asthma, atopy or myasthenia gravis; a history of allergy or sensitivity to acetylcysteine or contrast medium; current intake of prescription medicines; and a blood donation within the last 3 months. The same exclusion criteria applied for studies 2 and 4 except that patients with CKD stage III can have co-morbidity and take any prescription medicine with the exception of metformin. Metformin must be stopped on the day of the procedure and for two days following.

Our study is restricted to male volunteers. Previous experience has shown that regular voiding by female participants while receiving multiple infusions is difficult while maintaining volunteer privacy. Despite including only males, we do not know of any reason why the results will not be as relevant for women as they will be for men.

Participants can be withdrawn from the trial at their own request, at the request of the investigator in the context of safety concerns, or if the mean arterial pressure or heart rate increased by > 30 mmHg or > 30 bpm, respectively.

### Trial interventions

#### Acetylcysteine

There are currently no data to guide choice of an optimum oral or IV acetylcysteine regimen. This study will assess the effects of an IV and an oral dose of acetylcysteine (IV 200 mg/kg; oral 68.6 mg/kg in a 70 kg patient) that are similar to those currently used in clinical practice for the prevention of CIN. The oral dose is expected to produce a plasma concentration lower than the IV dose; however, a first pass effect after oral administration may allow effective conversion in the liver of acetylcysteine into cysteine and then glutathione, increasing the efficacy of the oral dose [29].

#### Selection of IV acetylcysteine dose

IV regimens have been assessed for prevention of CIN since they might be effective when started on the same day as contrast administration, rather than the day before [30]. The first trial used a dose similar to that used for early treatment of paracetamol-induced hepatotoxicity - 150 mg/kg over 30 min, then 50 mg/kg over 4 hrs (total dose 200 mg/kg) - and reported less nephropathy [10]. Other studies used lower doses (e.g. 500 mg over 15 mins [7.1 mg/kg in 70 kg patient] or 1000 mg twice, before and after the procedure, [28.5 mg/kg in 70 kg patient]) and did not find any benefit [30]. Overall, the choice of acetylcysteine regimen for previous studies seems to have been based on ease and prior practice in paracetamol poisoning rather than knowledge of acetylcysteine's effects on the kidney.

**Figure 2:**
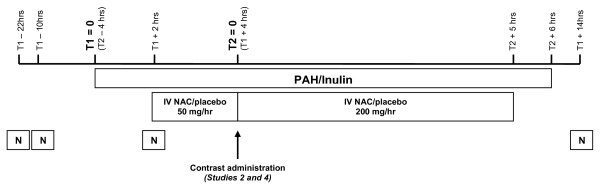
**Schema of study drug administration**. N, oral acetylcysteine.

We chose a revised IV high dose regimen (Figure 2) derived from pharmacokinetic data published by Prescott [31] after Monte Carlo simulations (Dr R Thanacoody, Royal Victoria Infirmary, Newcastle, unpublished) for two reasons. Firstly, the RCT that showed benefit with IV acetylcysteine used a high dose regimen similar to that used for paracetamol poisoning [23]. The subsequent negative studies used much lower doses, suggesting that IV acetylcysteine may need to be given in high doses. Secondly, the high peak plasma concentration that results from the rapid initial infusion of acetylcysteine for paracetamol poisoning produces nausea in about 40% of patients and anaphylactoid reactions in about 20% [32]. Although these reactions are routine and normally easily and safely treated by stopping the acetylcysteine infusion and administering antiemetic and antihistamine medications, it was important to reduce the rate of such reactions in study participants. The revised regimen provides a similar amount of acetylcysteine but administers it more evenly across 7 hours, producing a lower peak acetylcysteine concentration and a lower likelihood of nausea and anaphylactoid reactions.

The study uses the intravenous acetylcysteine preparation currently used in the Royal Infirmary of Edinburgh (Aurum Pharmaceuticals Ltd, Romford, UK) diluted in 5% dextrose solution. Placebo is 5% dextrose solution for infusion used in the Royal Infirmary of Edinburgh. Active or placebo infusions are prepared on each study day by clinical research facility nurses according to the randomized allocation order.

#### Selection of oral acetylcysteine dose

The dose used in oral regimens of acetylcysteine has ranged from 600 mg twice daily for 2 days, starting the day before radiocontrast administration (total dose 2.4 g, 34.3 mg/kg in a 70 kg patient) [23] up to 1500 mg twice daily for 2 days (total dose 6 g, 85.7 mg/kg in a 70 kg patient) [23]. Similar to more recent studies [12,13], we used 1200 mg (Figure 2) since there is some evidence that it is more effective than the more usually administered 600 mg [23].

Hard gelatin capsules containing acetylcysteine 600 mg or matched placebo (Lactose PhEur 600 mg) are prepared by Tayside Pharmaceuticals, Dundee (MA[IMP] #17859), and packaged in participant packs of 8 capsules. The capsules are delivered to the hospital pharmacy and supplied to participants according to the randomized allocation order. The active material and placebo capsule shells and raw material powders are obtained from an approved GMP supplier with appropriate TSE certificates. Capsules are filled by hand. Release testing for the capsules includes weight, appearance of contents, and product identification.

#### Contrast media

Participants in study 3 receive a single IV 100 ml dose of *Visipaque 320 *(iodixanol, equivalent to 320 mg iodine/ml), an iso-osmolar non-ionic radio-contrast agent, via a peripheral cannula [2]. We chose this dose because 100 mL is routinely used in the Royal Infirmary of Edinburgh for coronary angiography in CKD patients (Radiology Dept, personal communication). Larger doses of up to 400 mL are used if angioplasty is subsequently required. Such doses fall within the doses recommended in the summary of product characteristics [33], Martindale [34], and the literature [15]. The safety profile of such doses in healthy adults is excellent. In patients with CKD, iodixanol is at least as safe as other contrast agents [2]. Recent work suggests that neither CIN nor oliguria/need for dialysis will occur after administration of 100 mL of contrast to healthy participants with an eGFR of > 25.6 mL/min or > 64 mL/min, respectively [35].

Participants in study 4 received doses of *Visipaque 320 *as required by the consultant cardiologist doing the procedure to adequately visualise the coronary arteries and perform any procedure judged to be necessary.

### Study outcome measures

The primary outcome is a change in renal blood flow (RBF). Secondary outcomes include changes in GFR, tubular function, urinary proteins, and oxidative balance.

Changes in RBF after acetylcysteine and/or contrast administration are assessed by measuring plasma para-aminohippuric acid (PAH) clearance [36]. Change in RBF is the primary outcome since we expect this measure to be most sensitive to the effects of acetylcysteine and/or contrast administration. GFR is the best marker of global renal function. It can be directly measured using the ^51^Cr-EDTA method but this is a complicated technique and therefore we are using an alternative method by measuring plasma clearance of inulin [36].

Creatinine and cystatin C are measured in each participant. Plasma creatinine is measured by the hospital's clinical laboratory using validated methods. Urinary creatinine is measured by the picric acid method using a commercial kit (Alpha Laboratories, Ref 17609) in a 96-well plate. To 20 μL volumes of sample or standard solutions, 100 μL of Reagents 1 and 2 are added sequentially and the absorbance is measured at 510 nm on a plate reader immediately and after a further 6 min. The difference in absorbance values (6 min - 0 min) is used to calculate the results. Calibration is linear over the range 0-400 μg/mL of creatinine and samples are diluted with water as necessary to bring them within this range.

Serum cystatin C concentration may be a better marker of GFR than creatinine [37]. It is a small cysteine protease that is secreted at a fixed rate by all nucleated cells and is not affected by diet or muscle mass. In CIN, serum cystatin concentration peaks and normalizes more rapidly than creatinine [38]. Cystatin C is measured using a standard sandwich enzyme immunoassay (BioVendor Ref RD191009100) following manufacturers instructions. Plasma and urine samples are both diluted 1/400 before assay.

Kidney injury molecule-1 (KIM-1) and neutrophil gelatinase-associated lipocalin (NGAL) are measured in the urine of each participant. KIM-1 is a type I cell-membrane glycoprotein containing a unique six-cysteine immunoglobulin-like domain and mucin domain in its extracelluclar region [39]. Urinary KIM-1 has been shown to be an earlier diagnostic indicator of kidney injury when compared to conventional biomarkers such as creatinine [39]. NGAL is a protein bound to gelatinase in specific granules of neutrophils whose synthesis may be induced in epithelial cells in the setting of inflammation [40]. Animal [41] and human [42] studies have suggested that NGAL may also be powerful early biomarker of acute kidney injury. KIM-1 and NGAL are assayed using kits from R&D Systems (Cat N°.s DY1750 and DY1757 respectively) following the manufacturer's instructions with calibration ranges of 0-2500 pg/mL. Urine is assayed undiluted for KIM-1 and diluted 1 in 20 with water for NGAL.

Renal tubular function is assessed by measuring the kidney's fractional excretion of sodium [37,43]. Reductions in fractional excretion will supply information on renal perfusion and tubular function, and have been noted previously in CIN [44].

We are measuring plasma acetylcysteine and peripheral blood cell glutathione. Plasma aliquots are analysed by HPLC-FLD using an established method; briefly, samples are reduced with tributylphosphine, prior to protein precipitation using trichloroacetic and derivatization with 7-fluoro-benzo-2-oxa-1,3-diazole-4-sulfonate [45]. Derivatized samples are injected onto a C18 column (Gemini-NX) and eluted with 100 mM potassium phosphate:acetonitrile (94:6) at a flow rate of 1 mL/min prior to fluorescence detection (λex/λem = 385/515 nm). Peak areas are measured for quantitative calculations.

To quantify the thiol content in the buffy coat, 20 μL lysis buffer is added to 180 μL buffy coat samples and incubated at 4°C for 30 min, vortexing every 10 min. Albumin is removed from the buffy coat sample using an AlbuminOUT™ kit (G-Biosciences, St Louis, MO, USA). Protein content of the eluate is measured colorimetrically with an absorbance of 595 nm using the Bradford protein assay [46,47]. Samples are then analysed for thiol content using the same protocol as described above for plasma samples.

### Statistical analysis

Change in RBF is the primary outcome of the study. Mean RBF in healthy volunteers is 601 mL/min with a standard deviation of 114 mL/min [48]. A previous study [48] found the mean RBF in patients with CKD stage III to be 352 mL/min with a standard deviation of 104 mL/min. The cross-over studies, therefore, have an 80% power (alpha of 0.05) with n = 8 subjects to show a 16% change in RBF in healthy subjects and 33% change in patients with CKD.

The parallel group study has a 90% power (alpha of 0.05) with n = 22 subjects to show a 30% change in RBF in patients with CKD. Comparisons will be tested via a Student's *t*-test after ANOVA. Statistical significance will be taken at 5%.

### Ethics

Ethics approval was received from the Scotland A Research Ethics Committee, UK (reference number 07/MRE00/64).

## Discussion

Contrast agents are widely used for angiography and CT imaging. However, they are also associated with CIN, an important source of hospital morbidity and mortality. Although a number of preventative measures aimed at minimizing risk have been proposed, success has been partial due in part to the complex and poorly understood pathophysiology of CIN. Since the first clinical trial assessing acetylcysteine in the prevention of CIN was published in 2000 [49], there has been much debate over the degree of renoprotection offered by this drug [3,4,11,30].

While the use of acetylcysteine may be attractive due to ease of availability, familiarity of use amongst clinicians, and favourable side effect profile, the precise mechanism of action and appropriate dose and route of administration remain unclear. This has important implications for clinical practice as some clinicians informally report administering greater volumes of contrast media to those patients who have received acetylcysteine, in the belief that CIN will be prevented. This mechanistic trial should not only improve our understanding of CIN but may also lead to further dose response studies and clinical trials.

After initiating our study, two major meta-analyses were published that came to opposing views about the efficacy of acetylcysteine in CIN [50,51]. Furthermore, a recent large Brasilian RCT - ACT *Acetylcysteine for contrast-induced nephropathy *- reported that acetylcysteine 1200 mg oral twice daily for two days, starting the day before angiography, had no beneficial effect [12]. As a result, interest in acetylcysteine as a preventative treatment for CIN has fallen. However, like many previous studies, this study used a dose and route that was based on the original study [49] and not on any rational scientific basis. It therefore should not be used to discard acetylcysteine completely as a therapeutic option, in particular since one positive study used a much higher IV dose, similar to that administered to patients in one arm of our study. The results of our study may in fact show that IV, and not oral, acetylcysteine is required to have any effect on renal function.

Conducting a trial of this size with volunteers is complex and time consuming - there are over 132 study days lasting from 07.00 until early evening, together with 264 follow up visits. Shortly after commencing the study, the Scottish government introduced a directive to reduce waiting times for elective coronary angiography. Simultaneously, our hospital expanded its existing pilot primary angiography into a regional tertiary referral service for patients in three surrounding counties. As a result, there was a marked fall in the number of elective coronary angiographies taking place, as more patients underwent primary procedures. The elective coronary angiography waiting list was our principal source of potential volunteers for study 4 and so recruitment to study 4 was, and has remained, significantly slower than originally anticipated. We were unable to recruit patients undergoing primary angiographies due to the time needed for completion of the screening and consent process before the procedure. Only patients undergoing elective procedures were able to start oral acetylcysteine or placebo tablets 24 hours prior to their angiography. As a result, the study has taken many more months than initially anticipated.

We faced further difficulties obtaining PAH and inulin half way through the study, despite their use worldwide for many years as a standard method for assessing RBF and GFR. Since they are not medicines, and are not licensed for human use, we obtained them directly from the manufacturers. After commencing the trial, our sponsor deemed that these compounds had to be imported via an official importer rather than directly from the manufacturer. Due to importation regulations, obtaining permission for import was challenging, leading to a nine month delay in obtaining these compounds. Throughout this time, the study was put on hold.

Interest in the use of acetylcysteine to prevent CIN has fallen since the results of the ACT trial were announced. However, we believe that it is premature to discard this affordable, safe, and widely available medicine based on a lack of studies addressing the mechanisms of its effect, the best route of administration, and the correct dose. We hope that our study will ultimately allow the design of a large phase III study of NAC in CIN that uses a rational dose of NAC, given by the correct route, and assessed using the correct outcome measure.

## Competing interests

The authors declare that they have no competing interests.

## Authors' contributions

ME designed this study and wrote the grant application. DNB, DW, ILM, JG, and NU helped with the design of the RCT. ES and ME conducted all study visits, performed interim analysis and will carry out the final statistical analysis. SC, FC, SD, LB, JC, and JD were senior research nurses within the Wellcome Trust Clinical Research Facility and were integral in the setting up and running of the RCT. IM, AT, and NJ performed the assays. All authors read and approved the final manuscript.

## Pre-publication history

The pre-publication history for this paper can be accessed here:

http://www.biomedcentral.com/1472-6904/12/3/prepub
